# Exceptional dynamics at exceptional points

**DOI:** 10.1038/s41377-023-01347-4

**Published:** 2024-01-08

**Authors:** Wenjie Wan

**Affiliations:** 1 State Key Laboratory of Advanced Optical Communication Systems and Networks, University of Michigan-Shanghai Jiao Tong University Joint Institute, Shanghai Jiao Tong University, Shanghai, 200240 China; 2 Department of Physics and Astronomy, Shanghai Jiao Tong University, Shanghai, 200240 China

**Keywords:** Sub-wavelength optics, Photonic devices

## Abstract

Exceptional points (EPs), singularities of non-Hermitian systems, often exhibit exotic behaviors by engineering the balance between the system gain and loss. Now, EPs have been demonstrated to enable unidirectional perfect absorption/reflection at the visible light spectrum.

## Abstract (web summary)

Schematic view of the exceptional point on the bilayer metasurface, exhibiting a unidirectional scattering behavior of perfect absorption/reflection.

## Graphical abstract

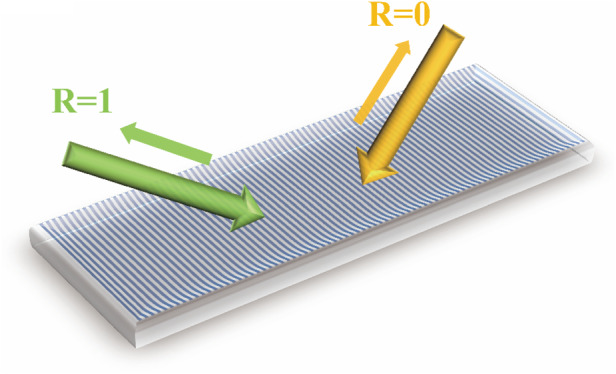

Observables in closed physical systems are commonly associated with Hermitian Hamiltonians in quantum theories. However, more general physical systems openly exchange particles and energy with their environment, rendering them non-Hermitian. Even though, non-Hermitian systems including representative examples like parity-time (PT) symmetry^1^ can show exotic and intriguing phenomena. Now, this emerging non-Hermitian physics has been widely investigated in many physical fields, including optics, acoustics, microwaves, electronics, opto-mechanics, and cold atoms. Essential to these findings is a prominent degeneracy called an exceptional point (EP), which corresponds to the phase transition point at which the eigenvalues of the underlying system and the corresponding eigenvectors simultaneously coalesce^2,3^. The abrupt nature of the EP leads to its strong response to external perturbations, enabling a hypersensitive sensing scheme for nanoparticles^4^, accelerometer^5^, and optical gyroscopes^6^. Moreover, exotic dynamics near an EP also reveal that the state evolution encircling an EP is found to be topological and chiral^7^. It is highly desirable to implement such EPs in the visible light regime, however, the required nano/micro fabrications to make such photonic structures are much more demanding.

Recently, in a newly published paper in *Light: Science & Applications*^8^, Xinbin Cheng from MOE Key Laboratory of Advanced Micro-Structured Materials, Tongji University, Shanghai, and Cheng-Wei Qiu from the National University of Singapore, Singapore, have jointly demonstrated such EP in the visible spectrum based on a bilayered metasurface, as illustrated in Fig. 1. By carefully engineering the gradient metagrating structures and tuning the interlayer loss, they showed a highly scattering EP by coupling the left and right incident light channels. Surprisingly, such metasurfaces reveal exotic behaviors of unidirectional perfect absorption/reflection right at the EP. This is caused by the asymmetrical design of the metasurface, unlike the traditional coherent perfect absorber without such asysmmetry^9^. While compared to prior works of unidirectional reflection induced by 1D PT-symmetric periodic structures^10,11^, the compact design and the perfect absorption make the current work more suitable for practical applications in all-optical information.

**Fig. 1:**
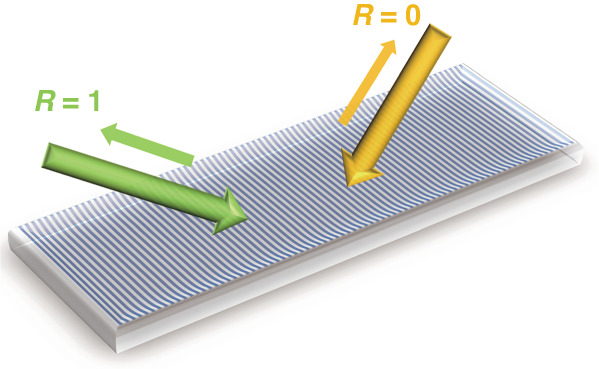
Schematic view of the exceptional point on the bilayer metasurface, exhibiting a unidirectional scattering behavior of perfect absorption/reflection

Finally, it is worth mentioning the potential of utilizing exceptional points can be of both practical and theoretical interest. Application-wise, the abrupt nature of the phase transition near the EP could be potentially beneficial for some ultrasensitive sensing schemes. The chirality encircling the EP could also be further explored for future topological devices. For the theoretical interests, there are two aspects of the EP: nonlinearity and quantum regimes, which are emerging quickly. For example, combining the nonlinearity around the EP may disturb the original chiral behaviors, leaving some nonlinear bistable states^12,13^. In the quantum case, it is a fascinating problem to show the single photon/biphoton quantum response right at the EP^14^, which still remains elusive. We envision future opportunities to exploit the singularity of EP with nonlinearity, and quantum for more exceptional work ahead, paving the way for their applications in all-optical processing, and quantum information.
